# Identification of Hemoglobin Levels Based on Anthropometric Indices in Elderly Koreans

**DOI:** 10.1371/journal.pone.0165622

**Published:** 2016-11-03

**Authors:** Bum Ju Lee, Jong Yeol Kim

**Affiliations:** KM Fundamental Research Division, Korea Institute of Oriental Medicine, Deajeon, Republic of Korea; Northeastern University, UNITED STATES

## Abstract

**Objectives:**

Anemia is independently and strongly associated with an increased risk of mortality in older people and is also strongly associated with obesity. The objectives of the present study were to examine the associations between the hemoglobin level and various anthropometric indices, to predict low and normal hemoglobin levels using combined anthropometric indices, and to assess differences in the hemoglobin level and anthropometric indices between Korean men and women.

**Methods:**

A total of 7,156 individuals ranging in age from 53–90 years participated in this retrospective cross-sectional study. Binary logistic regression (LR) and naïve Bayes (NB) models were used to identify significant differences in the anthropometric indices between subjects with low and normal hemoglobin levels and to assess the predictive power of these indices for the hemoglobin level.

**Results:**

Among all of the variables, age displayed the strongest association with the hemoglobin level in both men (p < 0.0001, odds ratio [OR] = 0.487, area under the receiver operating characteristic curve based on the LR [LR-AUC] = 0.702, NB-AUC = 0.701) and women (p < 0.0001, OR = 0.636, LR-AUC = 0.625, NB-AUC = 0.624). Among the anthropometric indices, weight and body mass index (BMI) were the best predictors of the hemoglobin level. The predictive powers of all of the variables were higher in men than in women. The AUC values for the NB-Wrapper and LR-Wrapper predictive models generated using combined anthropometric indices were 0.734 and 0.723, respectively, for men and 0.649 and 0.652, respectively, for women. The use of combined anthropometric indices may improve the predictive power for the hemoglobin level.

**Discussion:**

Among the various anthropometric indices, with the exception of age, we did not identify any indices that were better predictors than weight and BMI for low and normal hemoglobin levels. In addition, none of the ratios between pairs of indices were good indicators of the hemoglobin level. Finally, the Korean men tended to have higher associations between the anthropometric indices and anemia than the women.

## Introduction

Anemia is a significant disease in older men and women [1], and anemia and a low hemoglobin level have been independently and strongly associated with an increased risk of mortality in older people [1–3]. Anemia can occur for several reasons, including iron deficiency, renal disease, folate deficiency, nutritional deficiencies, and unexplained reasons [4]. The hemoglobin level is associated with aging because of its effects on the serum erythropoietin level and hematopoietic cell numbers [1, 5, 6] and obesity [1, 3, 4, 7–12]. Generally, the risk of iron deficiency anemia is higher in women than in men because of menstruation and other life stages in females [7, 13]. Additionally, the prevalence of anemia due to iron deficiency or low hemoglobin differs among different ethnic groups [8, 14]. For example, the risk of iron deficiency anemia is higher in Mexican-American women than in non-Hispanic white women [8].

Many studies conducted to date have focused on the association of the hemoglobin level with body mass index (BMI) or obesity. Several studies have suggested that anemia and the hemoglobin level are related to an increased BMI, a low activity level, an increased age, a low albumin level, a high creatinine level, stroke, gastric ulcers, low triglycerides, and malignant disease [1–4, 7–12]. For example, Izaks and colleagues [2] examined the relationship between mortality risk and anemia; this group used the World Health Organization (WHO) definition for older people. They demonstrated that anemia was associated with an increased mortality risk and that subjects with anemia had a high mortality rate in association with high rates of malignant disease and infection. However, although several studies have reported that a low hemoglobin level is strongly associated with obesity, almost all of these studies have focused only on the relationship between anemia and BMI, and prediction of the hemoglobin level using both individual and combined anthropometric indices has not yet been assessed.

Several recent studies have indicated the practical significance and/or utility of anthropometric indices, such as the waist circumference (WC), waist-to-hip ratio (WHR), and waist-to-height ratio (WHtR), in predicting the hemoglobin level in patients with different diseases [15–18]. Therefore, we hypothesized that anthropometric measurements other than weight and BMI may be potential indicators for predicting the hemoglobin level and that assessment of a combination of these measurements may provide enhanced predictive power with respect to the hemoglobin level. Thus, the present study aimed to address the following questions. Besides weight and BMI, which are well known to be associated with anemia, are any other specific anthropometric indices strongly associated with the hemoglobin level and anemia? If we identify additional anthropometric indices that are associated with anemia, then how accurately can we predict the hemoglobin level using a combination of these indices? Although several studies have suggested that anemia is associated with weight, BMI, the albumin and creatinine levels, and age, no study has examined whether the hemoglobin level can be predicted using a combination of various anthropometric indices, and no study has assessed the associations between the hemoglobin level and specific and diverse anthropometric indices.

The objectives of the present study were to examine the associations between the hemoglobin level and various anthropometric indices and to predict low and normal hemoglobin levels using combined indices. Furthermore, we examined differences in the hemoglobin level and anthropometric indices between Korean men and women. To our knowledge, this is the first report of associations between the hemoglobin level and anthropometric indices that includes a comparison of the predictive powers of individual and combined indices.

## Materials and Methods

### Subjects

A total of 7,156 subjects (4,073 women and 3,083 men) ranging in age from 53–90 years participated in this retrospective cross-sectional study. The subjects were recruited from several hospitals in Anseong, Ansan and other cities (27 urban and rural areas) in the Republic of Korea between November 2006 and August 2013. All of the study data were obtained from the Korean Health and Genome Epidemiology Study (KHGES) database and the Korea Institute of Oriental Medicine (KIOM). Written informed consent was obtained from all of the participants, and the KIOM Institutional Review Board approved this study (No. I-1210/002/002-02).

### Measurements and definitions

Blood samples were obtained from all subjects after an 8-hour fast by well-trained clinicians based on standardized protocols, and the hemoglobin levels were measured in all samples to detect the presence of anemia (ADVIA1800, Siemens, USA).

We obtained basic information, including gender, age, education level, and job status, by administering a questionnaire to the subjects. Based on standardized protocols, anthropometric indices, including weight, height, forehead, neck, axillary, chest, rib, pelvic, and hip circumferences and WC, were measured to the nearest 0.1 cm or 0.1 kg with the subject wearing lightweight clothing and no shoes. Non-elastic tape and equipment were used (LG-150; G Tech International Co., Ltd., Uijeongbu, Republic of Korea). We calculated BMI and the ratios between pairs of circumferences because these measurements have been used in many medical and epidemiological studies. The exact positions for the measurements of the human body and various indices used to calculate ratios are described in the references [19–22]. All of the indices assessed in this study are listed and briefly described in Table 1.

**Table 1:** Baseline characteristics and brief descriptions of all study variables.

	Men (n = 3,083)		Women (n = 4,073)		Description
Variable	Normal	Anemic	Normal	Anemic	
Participants	2,828	255	3,469	604	Number of participants
Weight^‡^	67.06 (9.441)	61.45 (9.892)	58.28 (8.2)	55.4 (8.678)	Weight
BMI^‡^	24.23 (2.893)	22.59 (3.035)	24.73 (3.18)	23.75 (3.313)	Body mass index
Age	62.46 (7.276)	67.99 (7.508)	62.75 (7.359)	66.23 (7.909)	Age
ForeheadC^‡^	56.49 (1.673)	56 (1.751)	54.71 (1.676)	54.5 (1.661)	Forehead circumference
NeckC^‡^	37.58 (2.42)	36.72 (2.621)	33.56 (2.165)	33.29 (2.13)	Neck circumference
AxillaryC^‡^	95.11 (5.654)	92.41 (5.805)	88.99 (5.872)	87.77 (6.122)	Axillary circumference
ChestC^‡^	93.85 (5.977)	91.39 (6.192)	92.75 (7.63)	91.08 (7.815)	Chest circumference
RibC^‡^	88.3 (6.309)	86.28 (6.847)	82.36 (7.754)	81.6 (7.876)	Rib circumference
WaistC^†^	87.79 (8.03)	84.99 (8.845)	87.19 (8.76)	85.88 (9.124)	Waist circumference (WC)
PelvicC^‡^	91.58 (6.141)	89.73 (6.415)	92.58 (6.851)	91.49 (6.823)	Pelvic circumference
HipC^‡^	92.83 (5.481)	90.79 (6.085)	93.46 (5.976)	92.16 (5.888)	Hip circumference
Forehead_Hip^‡^	0.61 (0.032)	0.619 (0.036)	0.587 (0.035)	0.593 (0.037)	Forehead-to-hip circumference ratio
Axillary_Hip^‡^	1.025 (0.043)	1.019 (0.047)	0.953 (0.045)	0.953 (0.047)	Axillary-to-hip circumference ratio
Waist_Hip^‡^	0.945 (0.055)	0.935 (0.063)	0.932 (0.066)	0.931 (0.07)	Waist-to-hip circumference ratio
Forehead_Pelvic^‡^	0.619 (0.038)	0.627 (0.04)	0.594 (0.043)	0.599 (0.045)	Forehead-to-pelvic circumference ratio
Axillary_Pelvic^‡^	1.04 (0.052)	1.032 (0.051)	0.963 (0.048)	0.961 (0.049)	Axillary-to-pelvic circumference ratio
Waist_Pelvic^‡^	0.958 (0.05)	0.946 (0.058)	0.941 (0.054)	0.938 (0.058)	Waist-to-pelvic circumference ratio
Forehead_Waist^‡^	0.648 (0.056)	0.665 (0.064)	0.633 (0.063)	0.641 (0.068)	Forehead-to-waist circumference ratio
Neck_Waist^‡^	0.43 (0.028)	0.435 (0.034)	0.387 (0.031)	0.391 (0.034)	Neck-to-waist circumference ratio
Chest_Waist^‡^	1.073 (0.059)	1.081 (0.071)	1.067 (0.06)	1.065 (0.063)	Chest-to-waist circumference ratio
Rib_Waist^‡^	1.009 (0.047)	1.019 (0.057)	0.947 (0.049)	0.953 (0.051)	Rib-to-waist circumference ratio
Forehead_Rib^‡^	0.642 (0.042)	0.652 (0.047)	0.67 (0.061)	0.674 (0.063)	Forehead-to-rib circumference ratio
Axillary_Rib^‡^	1.079 (0.043)	1.073 (0.045)	1.084 (0.056)	1.079 (0.055)	Axillary-to-rib circumference ratio
Forehead_Chest^‡^	0.604 (0.035)	0.615 (0.037)	0.593 (0.048)	0.602 (0.05)	Forehead-to-chest circumference ratio
Forehead_Axillary^‡^	0.596 (0.032)	0.608 (0.033)	0.617 (0.039)	0.624 (0.042)	Forehead-to-axillary circumference ratio
Forehead_Neck^‡^	1.508 (0.082)	1.531 (0.091)	1.636 (0.096)	1.642 (0.096)	Forehead-to-neck circumference ratio
WHtR^‡^	0.528 (0.048)	0.516 (0.05)	0.569 (0.061)	0.563 (0.062)	Waist-to-height circumference ratio
ASTS^‡^	28.01 (17.47)	27.62 (20.87)	25.46 (11.85)	23.87 (11.09)	Aspartate transaminase
ALTS^‡^	26.82 (15.37)	22.23 (11.02)	23.34 (14.93)	19.58 (11.09)	Alanine transaminase
BUNS^‡^	16.29 (4.31)	19.06 (8.481)	15.45 (4.035)	17.24 (5.501)	Blood urea nitrogen
CreatinineS^‡^	1.041 (0.146)	1.193 (0.597)	0.847 (0.117)	0.903 (0.351)	Creatinine
GluFBSS^‡^	104.5 (26.02)	108.1 (31.92)	101.2 (27.93)	102 (30.52)	Glucose
T.Chol^‡^	186.9 (33.71)	168.2 (35.09)	200.9 (35.42)	185 (33.93)	Total cholesterol
TgS^‡^	151.4 (107.8)	111.7 (56.74)	139.3 (76.21)	127 (74.56)	Triglycerides
HDL-C^‡^	43.85 (11.93)	42.34 (11.35)	48.04 (12.42)	45.01 (12.75)	High-density lipoprotein cholesterol
LDL-C^‡^	114.4 (32.48)	100.9 (31.63)	124.8 (32.98)	113.4 (30.69)	Low-density lipoprotein cholesterol
Hb^‡^	14.88 (0.996)	11.95 (1.07)	13.25 (0.751)	11.19 (0.894)	Hemoglobin
HCT^‡^	44.25 (3.114)	35.78 (3.16)	39.27 (2.374)	33.4 (2.574)	Hematocrit
SBP	123 (16.09)	123.8 (16.87)	122.5 (17.1)	123.1 (18.32)	Systolic blood pressure
DBP^‡^	79.75 (10.12)	74.63 (10.01)	78.17 (10.64)	75.42 (10.48)	Diastolic blood pressure

^‡^ p < 0.0001 ^†^ and p < 0.01 indicate a significant difference between the men and women. The gender differences calculated using the independent two-sample t-test were determined using non-transformed data.

Anemia was defined as a hemoglobin level of less than 12 g/dL for women and less than 13 g/dL for men (WHO, 1968); these diagnostic criteria for anemia have been used in many studies [1, 4, 5, 23]. Thus, male and female subjects meeting these WHO criteria were included in the anemia group.

### Statistical analysis

For statistical analysis, binary logistic regression (LR) was performed using SPSS 19 for Windows (SPSS Inc., Chicago, IL, USA) to identify significant differences between the normal hemoglobin group (1) and low hemoglobin group (0) after standardized transformation was applied to both the male and female data sets. For evaluation of odds ratios (ORs) and comparison of predictive power, standardization was applied to the age variable and all anthropometric indices. LR and naïve Bayes (NB) were applied using the Waikato Environment for Knowledge Analysis (WEKA) data mining tool [24] to assess the power of individual anthropometric indices to predict normal and low hemoglobin levels to obtain more reliable results.

For variable subset selection in the use of combined anthropometric indices, wrapper-based variable selection with a greedy stepwise search (forward) based on LR and NB was used to identify the optimal variable subsets, reduce the complexity of the model, and increase the predictive power. Wrapper-based variable selection methods [17, 25, 26] are powerful methods used to solve the problem of variable subset selection. These methods use the machine learning algorithm as a black box (induction algorithm) to evaluate subsets of variables according to predictive power and to detect the best subset. In other words, detailed information regarding the induction algorithm is not considered because it is only an interface. Searches performed using wrapper-based variable selection included 4 components: a state space, search method (i.e., best-first search or hill-climbing), initial state (i.e., forward selection or backward elimination), and termination condition. A state in the search space indicates a variable subset, and single variables were added or deleted by an operator. The termination condition is dependent on the search method used. The variable subset with the strongest predictive power in evaluation was selected as the final subset on which to run the induction algorithm [26].

Two prediction methods were used for each gender: the NB-Wrapper method (naïve Bayes with wrapper-based variable selection) and the LR-Wrapper method (logistic regression with wrapper-based variable selection). We utilized 10-fold cross-validation for validation of the models with individual and combined indices. We used the area under the receiver operating characteristic curve (AUC) as the main criterion in the comparison of predictive power because this value is generally applied to evaluate the predictive power of classifications or indices in medicine and biology. Additionally, we documented detailed results for each predictive model, including the sensitivity, 1-specificity, precision, and F-measure.

## Results

### Associations between the hemoglobin level and anthropometric indices

In the present study, the numbers of normal and anemic subjects based on their hemoglobin levels were 2,828 and 255 for men, respectively, and 3,469 and 604 for women, respectively. With regard to anthropometric indices, the predictive power of 27 variables for anemia was assessed, and all of them except for age showed significant differences between men and women. In addition, many of the variables exhibited significant differences between the normal and anemic groups in both men and women.

Tables 2 and 3 show the results of analyses of the associations between the various anthropometric indices and the hemoglobin level and the predictive power of each index in men and women. Among all of the variables examined for men, age displayed the strongest association with the hemoglobin level (p < 0.0001, OR = 0.487 [95% confidence interval, 0.428–0.555], LR-AUC = 0.702, NB-AUC = 0.701). Among the anthropometric indices in both the men and women, in addition to age, weight (p < 0.0001, OR = 1.87 [1.628–2.149], adjusted OR = 1.534 [1.329–1.772], LR-AUC = 0.663, NB-AUC = 0.661) and BMI (p < 0.0001, OR = 1.788 [1.562–2.046], adjusted OR = 1.537 [1.34–1.763], LR-AUC = 0.659, NB-AUC = 0.658) were the best indicators of anemia. Axillary circumference (AxillaryC) was also a useful predictor of the hemoglobin level (p < 0.0001, OR = 1.609 [1.412–1.835], adjusted OR = 1.406 [1.228–1.611], LR-AUC = 0.629, NB-AUC = 0.627).

**Table 2:** The associations between anthropometric indices and the hemoglobin level in men.

Index	Crude values		Age-adjusted values		AUC	
	p	OR	p	OR	LR	NB
Weight	< 0.0001	1.87 (1.628–2.149)	< 0.0001	1.534 (1.329–1.772)	0.663	0.661
BMI	< 0.0001	1.788 (1.562–2.046)	< 0.0001	1.537 (1.34–1.763)	0.659	0.658
Age	< 0.0001	0.487 (0.428–0.555)	-	-	0.702	0.701
ForeheadC	< 0.0001	1.343 (1.179–1.53)	0.0346	1.157 (1.011–1.325)	0.577	0.573
NeckC	< 0.0001	1.449 (1.267–1.658)	0.0006	1.271 (1.109–1.456)	0.602	0.596
AxillaryC	< 0.0001	1.609 (1.412–1.835)	< 0.0001	1.406 (1.228–1.611)	0.629	0.627
ChestC	< 0.0001	1.516 (1.329–1.729)	< 0.0001	1.397 (1.222–1.598)	0.613	0.611
RibC	< 0.0001	1.374 (1.208–1.564)	0.0001	1.298 (1.141–1.476)	0.582	0.574
WaistC (WC)	< 0.0001	1.41 (1.24–1.604)	< 0.0001	1.341 (1.182–1.522)	0.587	0.581
PelvicC	< 0.0001	1.356 (1.19–1.544)	0.0002	1.278 (1.123–1.455)	0.581	0.576
HipC	< 0.0001	1.46 (1.279–1.668)	0.0001	1.316 (1.152–1.505)	0.601	0.592
Forehead_Hip	< 0.0001	0.764 (0.673–0.868)	0.0003	0.788 (0.693–0.896)	0.571	0.56
Axillary_Hip	0.0299	1.151 (1.014–1.307)	0.3515	1.065 (0.933–1.217)	0.527	0.507
Waist_Hip (WHR)	0.0078	1.19 (1.047–1.353)	0.0012	1.232 (1.086–1.398)	0.547	0.542
Forehead_Pelvic	0.0026	0.825 (0.728–0.935)	0.0015	0.816 (0.72–0.925)	0.549	0.54
Axillary_Pelvic	0.0122	1.181 (1.037–1.344)	0.5086	1.046 (0.916–1.194)	0.547	0.546
Waist_Pelvic	0.0004	1.265 (1.111–1.44)	0.0006	1.252 (1.102–1.423)	0.563	0.562
Forehead_Waist	< 0.0001	0.757 (0.671–0.854)	< 0.0001	0.766 (0.68–0.862)	0.57	0.556
Neck_Waist	0.0118	0.851 (0.75–0.965)	0.0005	0.801 (0.708–0.907)	0.537	0.563
Chest_Waist	0.0414	0.878 (0.775–0.995)	0.0144	0.859 (0.761–0.97)	0.509	0.534
Rib_Waist	0.0009	0.814 (0.72–0.919)	0.0012	0.818 (0.724–0.924)	0.543	0.53
Forehead_Rib	0.0003	0.796 (0.703–0.902)	0.0003	0.794 (0.701–0.899)	0.558	0.545
Axillary_Rib	0.052	1.135 (0.999–1.29)	0.7868	0.982 (0.86–1.121)	0.533	0.519
Forehead_Chest	< 0.0001	0.739 (0.653–0.836)	< 0.0001	0.746 (0.657–0.846)	0.588	0.585
Forehead_Axillary	< 0.0001	0.707 (0.624–0.801)	< 0.0001	0.758 (0.67–0.858)	0.603	0.602
Forehead_Neck	< 0.0001	0.758 (0.667–0.861)	0.0017	0.811 (0.712–0.924)	0.578	0.57
WHtR	0.0001	1.295 (1.138–1.474)	< 0.0001	1.318 (1.162–1.495)	0.562	0.555

OR: odds ratio, AUC: area under the receiver operating characteristic curve, LR: logistic regression, NB: naïve Bayes. The crude AUC value was obtained. Statistical analyses shown in this table were performed using data transformed by standardization.

**Table 3:** The associations between anthropometric indices and the hemoglobin level in women.

Index	Crude value		Age-adjusted value		AUC	
	p	OR	p	OR	LR	NB
Weight	< 0.0001	1.439 (1.313–1.577)	< 0.0001	1.317 (1.201–1.444)	0.597	0.597
BMI	< 0.0001	1.376 (1.255–1.509)	< 0.0001	1.375 (1.254–1.507)	0.585	0.584
Age	< 0.0001	0.636 (0.583–0.693)			0.625	0.624
ForeheadC	0.0043	1.139 (1.042–1.246)	0.1907	1.063 (0.97–1.165)	0.535	0.534
NeckC	0.0051	1.135 (1.039–1.24)	0.0015	1.154 (1.056–1.262)	0.533	0.53
AxillaryC	< 0.0001	1.235 (1.13–1.349)	< 0.0001	1.217 (1.114–1.329)	0.557	0.555
ChestC	< 0.0001	1.247 (1.142–1.363)	< 0.0001	1.268 (1.16–1.386)	0.561	0.56
RibC	0.0261	1.105 (1.012–1.206)	< 0.0001	1.207 (1.103–1.321)	0.527	0.527
WaistC (WC)	0.0008	1.162 (1.065–1.269)	< 0.0001	1.296 (1.184–1.418)	0.537	0.534
PelvicC	0.0003	1.176 (1.076–1.285)	< 0.0001	1.223 (1.119–1.338)	0.537	0.536
HipC	< 0.0001	1.257 (1.148–1.377)	< 0.0001	1.219 (1.113–1.334)	0.559	0.559
Forehead_Hip	0.0001	0.843 (0.773–0.92)	0.0001	0.839 (0.768–0.916)	0.542	0.541
Axillary_Hip	0.996	1 (0.917–1.09)	0.6607	1.02 (0.935–1.112)	0.474	0.482
Waist_Hip (WHR)	0.6504	1.02 (0.936–1.112)	< 0.0001	1.23 (1.122–1.349)	0.498	0.515
Forehead_Pelvic	0.0102	0.894 (0.82–0.974)	< 0.0001	0.832 (0.763–0.908)	0.523	0.515
Axillary_Pelvic	0.3296	1.044 (0.957–1.139)	0.5042	0.971 (0.89–1.059)	0.512	0.496
Waist_Pelvic	0.1615	1.064 (0.976–1.16)	< 0.0001	1.218 (1.114–1.333)	0.51	0.509
Forehead_Waist	0.0044	0.884 (0.812–0.962)	< 0.0001	0.777 (0.712–0.848)	0.526	0.52
Neck_Waist	0.0209	0.904 (0.83–0.985)	< 0.0001	0.807 (0.74–0.881)	0.516	0.506
Chest_Waist	0.3219	1.045 (0.958–1.139)	0.0101	0.886 (0.808–0.972)	0.515	0.513
Rib_Waist	0.0061	0.887 (0.815–0.967)	0.0002	0.849 (0.779–0.925)	0.531	0.53
Forehead_Rib	0.1438	0.938 (0.86–1.022)	0.0001	0.835 (0.764–0.913)	0.516	0.517
Axillary_Rib	0.0408	1.095 (1.004–1.194)	0.0655	0.915 (0.833–1.006)	0.525	0.519
Forehead_Chest	< 0.0001	0.834 (0.766–0.909)	< 0.0001	0.798 (0.731–0.87)	0.551	0.548
Forehead_Axillary	0.0002	0.849 (0.78–0.926)	< 0.0001	0.834 (0.765–0.909)	0.543	0.536
Forehead_Neck	0.1166	0.933 (0.856–1.017)	0.0052	0.882 (0.808–0.963)	0.516	0.511
WHtR	0.0437	1.094 (1.003–1.194)	< 0.0001	1.318 (1.201–1.448)	0.52	0.516

OR: odds ratio, AUC: area under the receiver operating characteristic curve, LR: logistic regression, NB: naïve Bayes. The crude AUC value was obtained. Statistical analyses shown in this table were performed using data transformed by standardization.

In women, as in men, age showed the strongest association with the hemoglobin level among the studied variables (p < 0.0001, OR = 0.636 [0.583–0.693], LR-AUC = 0.625, NB-AUC = 0.624). Among the anthropometric indices in both men and women, weight (p < 0.0001, OR = 1.439 [1.313–1.577], adjusted OR = 1.317 [1.201–1.444], AUC = 0.597) and BMI (p < 0.0001, OR = 1.376 [1.255–1.509], adjusted OR = 1.375 [1.254–1.507], LR-AUC = 0.585, NB-AUC = 0.584) displayed the strongest associations with anemia. None of the ratios between pairs of indices were good indicators of the hemoglobin level.

Analysis of gender differences revealed that age was the strongest indicator of anemia and that weight and BMI were the best anthropometric predictors in both men and women. However, the significance of the differences and predictive powers of all of the variables were greater in men than in women.

### Comparison of the predictive powers of the combined anthropometric indices

Table 4 presents the results of the prediction models constructed using combined anthropometric indices based on the NB-Wrapper and LR-Wrapper methods. The AUC values for the NB-Wrapper and LR-Wrapper methods were 0.734 and 0.723, respectively, for men and 0.649 and 0.652, respectively, for women. Compared with the AUC values for weight, which was the best individual indicator of anemia for both the men and women, the AUC values for the combined indices revealed slight increases of 0.073 and 0.06 for the NB-Wrapper and LR-Wrapper methods, respectively, for men and 0.052 and 0.055, respectively, for women. Table 5 lists the indices selected by wrapper-based variable subset selection for each model. For example, the model created using the NB-Wrapper method for men included anthropometric indices such as BMI, age, axillary-to-pelvic circumference ratio (Axillary_Pelvic), neck-to-waist circumference ratio (Neck_Waist), and axillary-to-rib circumference ratio (Axillary_Rib). The results indicated that although BMI and weight were the best predictors of anemia, the use of combined indices could improve the predictive power of the model. In detailed performance analysis, groups with relatively large numbers of subjects had better predictive powers than those with relatively few subjects because the class imbalance problem occurred in all of the models. For example, for men, the sensitivities of the NB-Wrapper method were 0.987 and 0.047 in the normal and anemic groups, respectively, whereas the 1-specificity values were 0.953 and 0.013, respectively. The main reasons for this problem were the low prevalence of subjects with a low hemoglobin level and the fact that most of the classification algorithms were designed for performing calculations for groups with similar sample sizes [19–21, 27–29]. The class imbalance problem may be solved using under- and over-sampling or synthetic sampling techniques, but the use of such sampling techniques may lead to a loss of data, such as the prevalence in the original data set. Therefore, we used the original data set to more accurately reflect the characteristics of the medical data.

**Table 4:** Analysis of the predictive powers of the four models constructed using combined variables.

Gender	Method	Class	Sensitivity	1-specificity	Precision	F-measure	AUC
Men	NB-Wrapper	Normal	0.987	0.953	0.92	0.952	0.734
		Anemic	0.047	0.013	0.245	0.079	
	LR-Wrapper	Normal	0.986	0.957	0.92	0.952	0.723
		Anemic	0.043	0.014	0.216	0.072	
Women	NB-Wrapper	Normal	0.997	0.99	0.853	0.919	0.649
		Anemic	0.01	0.003	0.353	0.019	
	LR-Wrapper	Normal	0.999	0.995	0.852	0.92	0.652
		Anemic	0.005	0.001	0.5	0.01	

NB-Wrapper: naïve Bayes with wrapper-based variable subset selection technique, LR-Wrapper: logistic regression with wrapper-based variable subset selection technique, AUC: area under the receiver operating characteristic curve. Statistical analyses shown in this table were performed using data transformed by standardization. Detailed classification performance results are grouped by class (normal and anemic groups) using a confusion matrix. For example, for the NB-Wrapper method for men, the sensitivity and 1-specificity for each class were calculated using the following formulas: sensitivity = TP/(TP+FN); and 1-specificity = FP/(TN+FP) = 1-TN/(TN+FP) for the normal group; and sensitivity = TN/(TN+FP) and 1-specificity = FN/(TP+FN) = 1-TP/(TP+FN) for the anemic group.

**Table 5:** Variables selected using the variable selection technique for each model.

Gender	Method	Num. of variables	Variables
Men	NB-Wrapper	5	BMI, Age, Axillary_Pelvic, Neck_Waist, Axillary_Rib
	LR-Wrapper	4	BMI, Age, Axillary_Rib, WHtR
Women	NB-Wrapper	3	BMI, Age, Rib_Waist
	LR-Wrapper	5	BMI, Age, PelvicC, Axillary_Hip, Rib_Waist

NB-Wrapper: naïve Bayes with wrapper-based variable subset selection technique, LR-Wrapper: logistic regression with wrapper-based variable subset selection technique.

## Discussion

In this study, we examined the associations between various anthropometric indices and the hemoglobin level, as well as the predictive powers of individual and combined indices, using a computational approach in Korean men and women.

Previous studies have indicated the practical significance and/or utility of anthropometric indices such as the WC, WHR, and WHtR in predicting the hemoglobin level in patients with different diseases [15–18]. For instance, Odagiri et al. [15] assessed the associations among the WHtR, hemoglobin level, and chronic kidney disease. They reported significant associations between the WHtR and the hemoglobin level, BP, HDL cholesterol, LDL cholesterol, and hematocrit and identified significant positive associations between quartiles of the WHtR and the hemoglobin, hemoglobin A1c, and fasting plasma glucose levels, BP, and hematocrit. In addition, this group reported that anemia was likely predictive of the progression of chronic kidney disease because a low hemoglobin level can exacerbate hypoxia. Further, they demonstrated that the WHtR was an independent predictor of chronic kidney disease. Vuong et al. [16] have reported that WC is a strong indicator of hematological parameters such as the hemoglobin level, red blood cell count, mean cell hemoglobin, and iron level and that BMI exhibits weaknesses in terms of patient and physician understanding. They documented positive correlations between WC and the hemoglobin level, hematocrit, and red blood cell count; in addition, they found that an increased hemoglobin level was associated with increased risks of ischemic stroke and chronic hypoxia. Furthermore, this group revealed that the hemoglobin levels in a red blood cell and in a volume of packed red blood cells decrease with increasing WC. Further, Al-Hashem et al. [17] assessed the practical significance of the associations between BMI and the hemoglobin level and between WC and the hemoglobin level for diagnosing anemia and found that the mean hemoglobin level was highly positively and significantly associated with WC but that it was not significantly associated with BMI. This group indicated that WC should be used instead of BMI to predict the hemoglobin level and to screen for anemia based on this level. Moreover, Lee et al. [18] measured the hemoglobin concentrations in high school students (99 males and 116 females) and found that nearly half of them had anemia. Furthermore, these authors argued that the hemoglobin concentration was more strongly positively correlated with height and the WHR than with weight and BMI. However, in our study, the associations of the hemoglobin level with WC and the WHtR were lower than those of the hemoglobin level with BMI and weight, in contrast with previous studies.

Several studies have investigated the associations of anemia with gender and age. Neymotin and colleagues [7] and Takeda and colleagues [13] have reported that the risk of iron deficiency anemia is higher in women than in men because of menstruation and other life stages in females. Conversely, Timiras and colleague [30] have reported that the prevalence of anemia is higher in men than in women between the ages of 60 and 96 years. Further, Guralnik and colleagues [14] have reported that the prevalence of anemia is much lower in men than in women who are middle aged or younger (less than 49 years) but that this prevalence is much higher in men than in women of older ages (75 years and older). The authors indicated that their findings may have been attributed to differences in the anemia criteria recommended by the WHO for women (12 g/dL) and men (13 g/dL). Using the same criteria for both men and women, they found that the prevalence of anemia in women aged 65 years and older was higher than that in men of the same age. Notably, increased age has been reported to be a major risk factor for anemia [1, 4]. Timiras and colleagues [30] demonstrated that the hemoglobin levels in healthy elderly subjects with an average age of 70 years did not change substantially with increasing age, in contrast with the levels in men over the age of 85 years. However, Salive and colleagues [31] have reported that anemia and the hemoglobin level are strongly associated with the aging process, indicating that the association between the hemoglobin level and aging is stronger in men than in women. In addition, Guralnik and colleagues [14] have demonstrated that the prevalence of anemia is significantly increased with increasing age in both men and women. Our results are in agreement with those of previous studies [1, 4, 14, 31]. Our data indicated that age was the most strongly associated with the hemoglobin level in both men and women and that this association was higher in men than in women. Furthermore, the associations between the anthropometric indices and the hemoglobin level were lower in women than in men.

Although previous studies have revealed several associations between other diseases and ratio indices, such as the WHR and WHtR, which are commonly used in epidemiology and medicine and are closely associated with hypertension [20], hypertriglyceridemia [21, 32], hyper-HDL and LDL cholesterolemia [33, 34], and type 2 diabetes [19, 35], in this investigation, the ratio indices were not as useful as BMI and weight for predicting the hemoglobin level or anemia. In our study, we found that the ratio indices and WC exhibited weaker predictive powers for anemia than weight and BMI in both men and women.

The association between obesity and anemia has been studied in several countries [1–4, 7–12]. In the US [3, 4, 7, 8, 10], Zakai and colleagues [3] have reported that a low BMI, low activity level, and diseases such as frailty, congestive heart failure, and stroke are the best predictors of a low hemoglobin level. Further, Semba and colleagues [4] reported no or a weak association between BMI and anemia but that subjects with anemia due to renal disease comprised the largest percentage of those with a low BMI. Additionally, Ausk and colleagues [10] reported that obese and overweight subjects were less likely to be anemic than normal subjects but that an increased BMI was not associated with anemia related to chronic inflammation. In Italy, Cesari and colleagues [1] observed that anemic subjects were older and had lower BMIs and educational levels, fewer strokes and gastric ulcers, and increased serum creatinine levels. They also observed decreased triglyceride levels, total cholesterol levels, and albumin levels among the anemic subjects. In China, Qin and colleagues [11] observed that females with abdominal obesity were less likely than those with normal weight to have a low hemoglobin level; they also found that obese females with a high BMI had a higher hemoglobin level than underweight, normal and overweight females. In Mexico, Cepeda-Lopez and colleagues [12] reported that the risk of iron deficiency was higher in obese females and children than in normal subjects under similar conditions with similar dietary iron intake. This group indicated that obesity-related inflammation and dietary iron absorption most likely led to the increased risk of iron deficiency. In Korea, Choi and colleagues [9] conducted a prospective study to examine the clinical characteristics of anemia in older Koreans and reported that the independent risk factors included an older age, a low BMI, a low albumin level, a higher creatinine level, and the female gender. Our results are consistent with those of previous studies [1, 3, 4, 9, 11]. In our study, BMI was determined to be one of the best predictors of low and normal hemoglobin levels.

Several previous studies [1, 3, 9, 11] have suggested that weight and BMI are strongly associated with the hemoglobin level and anemia. Although we did not identify any anthropometric indices that were better predictors of the hemoglobin level than weight and BMI, our predictive models, which were constructed using a supervised machine learning technique and a combination of various anthropometric indices, slightly increased the predictive power for the hemoglobin level compared with those of weight, BMI, and age, which had the highest individual predictive powers. In epidemiological or large-scale health surveys, identification or prediction of the hemoglobin level using anthropometric indices represents a cost-effective and easy method (non-invasive measurement) compared with blood tests (invasive measurement). Therefore, our methods might enable anemia prediction in remote healthcare or telemedicine.

In conclusion, we did not identify any anthropometric indices, with the exception of age, that were better predictors than weight and BMI for low and normal hemoglobin levels among the various indices studied. Among all of the anthropometric indices, weight and BMI showed the strongest associations with the hemoglobin level and displayed the best predictive powers. Age was the most strongly associated with the hemoglobin level in both men and women, and this association was higher in men than in women. Additionally, the associations between the anthropometric indices and the hemoglobin level were lower in women than in men. Furthermore, the use of combined anthropometric indices improved the predictive power for diagnosing anemia compared with the use of weight or BMI. Due to the cross-sectional design of this study, the causal relationship between the hemoglobin level and BMI or weight could not be assessed. Another limitation of this study was the class imbalance problem caused by the small number of subjects with anemia.
